# Perioperative cerebrospinal fluid drainage for the prevention of spinal ischemia after endovascular aortic repair

**DOI:** 10.1007/s00772-017-0261-z

**Published:** 2017-05-16

**Authors:** M. Wortmann, D. Böckler, P. Geisbüsch

**Affiliations:** Klinik für Gefäßchirurgie und Endovaskuläre Chirurgie, Universitätsklinikum Heidelberg, Im Neuenheimer Feld 110, 69120 Heidelberg, Germany

**Keywords:** Spinal ischemia, Cerebrospinal fluid drainage, Thoracic endovascular repair, Thoracic aortic disease, Thoracoabdominal aortic disease, Spinale Ischämie, Liquordrainage, Endovaskuläre Therapie, Thorakale Aortenerkrankung, Thorakoabdominelle Aortenerkrankung

## Abstract

Endovascular treatment of thoracic and thoracoabdominal aortic diseases is accompanied by a risk of spinal ischemia in 1–19% of patients, depending on the entity and extent of the disease. The use of perioperative drainage of cerebrospinal fluid is one of the invasive measures to reduce the occurrence of this severe complication. This article reviews the incidence of spinal ischemia, its risk factors, the evidence for carrying out cerebrospinal fluid drainage and its modern use by means of an automated, pressure controlled system (LiquoGuard®7).

## Zusammenfassung

Die endovaskuläre Therapie von thorakalen und thorakoabdominellen Aortenerkrankungen geht, abhängig von der Entität und Ausprägung der jeweiligen Erkrankung, mit einem Risiko von 1–19 % für eine spinale Ischämie einher. Der Einsatz einer perioperativen Liquordrainage ist eine der invasiven Maßnahmen zur Verringerung des Auftretens dieser schwerwiegenden Komplikation. Diese Übersichtsarbeit legt die Inzidenz spinaler Ischämien, die Evidenz zur Durchführung einer perioperativen Liquordrainage und deren moderne Anwendung in Form eines automatisierten, druckkontrollierten Systems (LiquoGuard®7) dar.

## Introduction

Spinal ischemia represents one of the most serious complications in the treatment of thoracic and thoracoabdominal aortic pathologies. The neurological deficits in the form of paraparesis, paraplegia, or urine and fecal incontinence that can occur in the course of spinal ischemia dramatically reduce quality of life. In addition, affected patients suffer from a significantly increased mortality in the postoperative course [1]. Besides age and the postoperative acute kidney failure, paraplegia is also one of the most important predictors for mortality [2].

Whereas the incidence of spinal ischemia is around 20% in open procedures, endovascular treatment of the same pathologies is associated with a significantly reduced risk for neurological complications [3]. This risk ranges from 1.2 to 8% in the literature [4–9]. However, it can increase to 19% in subgroups, e. g., patients with Crawford II aneurysms [10].

A multitude of invasive and noninvasive perioperative measures have been developed for the open surgical management of thoracic and thoracoabdominal aortic disorders in order to reduce the incidence of spinal ischemia and minimize the resultant neurological deficits. Examples include strict avoidance of hypotensive phases during surgery and the postoperative phase, reimplantation of large intercostal arteries, permissive hypothermia, and catheter placement for the perioperative drainage of cerebrospinal fluid (CSF). By consistently implementing these protective measures, it is possible to reduce the rate of neurological complications to 5% at specialized centers [11].

Although many of these measures can only be employed during open procedures, some, including perioperative CSF drainage, can also be performed during endovascular interventions. Table 1 summarizes the measures most commonly used to reduce spinal ischemia in endovascular procedures.

**Table 1:** Possible measures to avoid spinal ischemia

Measures to prevent and treat spinal ischemia	References
Prevention of hypotensive phases (mean arterial pressure >90 mm Hg)	[12, 13]
Shortest possible treatment duration	[14, 15]
Staged approach in complex endovascular procedures	[16, 17]
Preservation of perfusion to the subclavian artery; where necessary, revascularization if stent coverage is planned	[18, 19]
Preservation of perfusion to the internal iliac arteries	[19, 20]
Drainage of cerebrospinal fluid	[12, 13, 21]
Local or systemic hypothermia	[22]^a^; [23]
Optimization of hemoglobin levels	[24]
Preoperative coiling of lumbar arteries	[25]^b^; [26]
Neurophysiological monitoring	[21, 27, 28]
Drug therapy (e. g., intrathecal papaverine)	[29]^a^; [30]^a^

^a^Citation relates to open surgical treatment

^b^Citation relates to experimental work

This article provides an overview of the current data on the employment of perioperative CSF drainage in the endovascular treatment of thoracic and thoracoabdominal aortic pathologies. In addition to the current guideline recommendations, the article also discusses the risks, the different implementation protocols, and our center-specific guidelines for the use of an automated, pressure-controlled CSF drainage system (*LiquoGuard®7*, Möller Medical GmbH, Fulda, Germany).

## Incidence of and risk factors for spinal ischemia

Persistent neurological deficits due to spinal ischemia resulting from endovascular treatment are seen in about 2–8% of cases [4, 8–10, 31]. Transient neurological deficits, on the other hand, are significantly more frequently reported at an incidence of up to 20% [6].

Persistent neurological deficits are seen in about 2–8% of cases

The main risk factor for spinal ischemia is the length and localization of the aortic segment to be treated [10, 32]. Yet, there is still controversy regarding the length of aortic segment treated as an independent risk factor following endovascular treatment. For example, the risk of spinal ischemia in an open surgical repair for Crawford I and II aneurysms is as high as 38%. In the case of Crawford III or IV aneurysms, the risk is significantly lower at 12% [32]. Comparably, the incidence of spinal ischemia in Crawford II aneurysms is the highest at up to 19% after endovascular aortic repair. This is followed in descending order by Crawford I, III, and IV aneurysms [10]. In a case series of 142 patients who underwent endovascular treatment for thoracoabdominal aneurysm, the length of the aortic segment treated was the only significant risk factor for the occurrence of spinal ischemia [33]. This can be explained by the increased number of intercostal or lumbar arteries covered, resulting in reduced perfusion of the collateral network. In this regard, the aorta at the Th9–Th12 level is considered the most critical segment, since the arteria radicularis magna (artery of Adamkiewicz) arises in this region as an unpaired vessel that plays a key role in the perfusion of the spinal cord. A further reduction of collateral flow, e. g., due to previous infrarenal aortic repair or occlusion of the left subclavian artery or the internal iliac arteries, also increases the risk for neurological complications. Table 2 provides an overview of the risk factors for spinal ischemia.

**Table 2:** Risk factors for the occurrence of spinal ischemia

Risk factors	References
Long aortic lesions/long aortic coverage (>20 cm)	[10, 18, 34, 35]
Prior aortic surgery (e. g., abdominal aortic repair)	[10, 14, 16]
Stent placement at the level of the visceral segment Th9–Th12 (origin of the arteria radicularis magna [artery of Adamkiewicz])	[34]
Coverage of the left subclavian artery	[18]
Occlusion of the internal iliac arteries	[20]
Chronic renal insufficiency	[18, 36]
Perioperative hypotension	[37]
Female gender	[35]
Long procedure time	[36]

In the majority of cases, neurological complications emerge within the first 2–3 postoperative days [38, 39]. However, a small number of patients develop neurological symptoms only after several weeks despite an initially complication-free course [6, 38].

## Evidence for the use of perioperative cerebrospinal fluid drainage in endovascular procedures

A study using an animal model showed that CSF drainage can lower the incidence of spinal ischemia following thoracic aortic procedures and reduce the severity of neurological deficits [39]. The efficacy of perioperative CSF drainage in open thoracic aortic repair in humans has been investigated in three randomized trials [40–42].

The first of these three studies failed to achieve a reduction in the rate of neurological complications. However, given our current knowledge, this can be explained by inadequate employment of CSF drainage at a drainage volume of only 50 ml CSF per procedure and the resultant inadequate reduction in intracranial pressure [40]. Two other randomized studies showed a significant protective effect for perioperative CSF drainage with a reduction in the rate of spinal ischemia of up to 80% [41, 42], whereby papaverine was additionally administered in one of these two studies [41]. In addition to these three randomized studies, there are several meta-analyses and systematic reviews demonstrating that CSF drainage reduces the rate of spinal ischemia in open surgical procedures [43–45].

The extent to which this evidence can be extrapolated to the endovascular management of aortic disease is as yet unclear [43]. Having said that, there is an increasing number of case series that describe a reduction in the risk of spinal ischemia also during endovascular procedures as a result of the use of CSF drainage [12].

A systematic review of almost 5000 patients put the incidence of spinal ischemia in patients receiving routine perioperative CSF drainage at 3.5%. Thus, no benefit was seen in comparison to patients not receiving drainage, in which spinal ischemia occurred in 3.2% of cases [9].

If neurological deficits due to spinal ischemia have already emerged in the postoperative period, a combination of spinal catheter placement for CSF drainage and raising mean arterial pressure represents an effective treatment option. This results in a complete resolution of neurological deficits in 30–90% of cases [1, 46–48].

## Guideline recommendations on perioperative cerebrospinal fluid drainage in endovascular procedures

Due to the lack of evidence, the current guidelines provide no clear recommendation on the use of CSF drainage in complex endovascular aortic procedures such as TEVAR, FEVAR, or BEVAR. According to the current guideline of the European Society of Cardiology, the use of CSF drainage can be considered in patients at increased risk for spinal ischemia (Class IIa C) [49].

The position paper published in 2015 by the European Association for Cardio-Thoracic Surgery makes the same recommendation (Class IIa C) [50].

The interdisciplinary guideline issued by several American medical societies recommends perioperative CSF drainage in patients at increased risk for spinal ischemia in both open and endovascular procedures (Class I B), although only references relating to the use of CSF drainage in open repair are cited at the respective section [51].

The 2010 German Society for Vascular Surgery (DGG) guideline makes no recommendation on CSF drainage in endovascular procedures [52].

## Implementation protocols for cerebrospinal fluid drainage

There are three different feasible protocols for the implementation of perioperative CSF drainage:

1. In the case of routine implementation, all patients undergo preoperative spinal catheter placement prior to the planned endovascular procedure [12].
2. In a so called selective implementation, only those patients at increased risk for spinal ischemia undergo preoperative spinal catheter placement. This is intended to reduce the incidence of neurological complications in high-risk patients without exposing those patients at low risk to the potential additional complications associated with spinal catheter placement. Selective use currently represents the most widespread implementation protocol for perioperative CSF drainage in endovascular procedures [2, 4, 46, 47]. However, there are no standardized recommendations on patient selection or on the precise conduction of the CSF drainage.
3. The third possible is to entirely waive a preoperative placement of a spinal catheter. Only those patients who develop spinal ischemia postoperatively undergo emergency spinal catheter placement. In such cases, a consistent reduction in CSF pressure is able to achieve a significant improvement in neurological complications [1]. However, neurological deficits persist in up to 30% of patients despite this intervention.

## Complications of cerebrospinal fluid drainage

The most serious complication associated with perioperative CSF drainage is cerebral hemorrhage in the form of intracerebral, subarachnoid, or subdural hemorrhage. While the majority of patients sustain either no or only mild neurological deficits as a result of cerebral hemorrhage, cases of severe neurological complications, as well as fatal outcomes, have also been described [47, 48]. Larger volumes of CSF drainage appear to be an independent risk factor for bleeding [48]. In a case series with 230 patients, for example, the 8 patients that developed subdural hematoma exhibited a mean drainage volume of 690 ml. Thus, this drainage volume was significantly higher compared with patients not affected by this complication, in whom a mean volume of 360 ml CSF was drained [53]. Moreover, the authors of that particular study did not recommend reducing intracranial pressure (ICP) to below 8 mm Hg, since a low ICP also correlated with the occurrence of intracerebral hemorrhage. This, however, could not be confirmed in other studies [48].

If bloody fluid is seen through the spinal catheter, CSF drainage should be stopped immediately followed by optimization of blood coagulation, removal of the spinal catheter and spinal as well as cerebral imaging. Since patients with pre-existing neurological conditions or coagulation disorders are at greater risk for bleeding, they should undergo cranial CT prior to spinal catheter placement and the indication for the use of CSF drainage should be made with great caution [24].

Due to the large diameter of the spinal catheter used, with an outer diameter of up to 1.6 mm, the rate of postdural puncture headaches and CSF leakage requiring treatment is between 3 and 20% and, as such, relatively high [47, 48].

Intracerebral hemorrhage represents the most serious complication

At a rate of 1%, infections in the form of meningitis due to spinal catheters being in situ for several days are very rare [48, 54].

A large case series of over 1000 patients who received a spinal catheter for perioperative CSF drainage reported a 99.8% technical success rate for drainage placement. Complications were seen in 1.5% of cases, whereby subdural hematoma was identified in 0.4% of all patients [54].

In summary, the incidence of serious complications due to spinal catheter placement, as well as perioperative CSF drainage itself, is between 1 and 4% [46, 47], while the fatality rate is around 1% [48]. The rate of minor complications, however, is significantly higher, being reported at as much as 30% in few series [47].

## The Heidelberg algorithm

At our clinic, we perform SOP-based selective placement of CSF drainage (see point 2 under “Implementation protocols for cerebrospinal fluid drainage”). The criteria for this include, e. g., long segment of aorta to be treated (>20 cm), prior infrarenal surgery (open or endovascular), occlusion of the internal iliac arteries or the left subclavian artery [47]. Patients undergoing treatment due to aortic rupture do not receive a spinal catheter preoperatively. If spinal ischemia should develop postoperatively in such cases, a spinal catheter is immediately placed and CSF drainage initiated. In the case of impaired coagulation or previous spinal surgery, further diagnostics are initiated in close consultation with our anesthesiology colleagues. In these patients, the further procceding is determined according to the individual risk profile as a case by case decision.

Spinal catheters are placed on the day prior to surgery. This has proved practical compared with placement directly prior to of surgery since, particularly in the case of complex endovascular procedures, sufficient heparin administration is required; this, however, should be avoided in the first hour following catheter placement. As in the “Guidelines on neuraxial regional anesthesia and thromboembolism prophylaxis/antithrombotic medication” issued by the German Society for Anesthesiology and Intensive Medicine, antiplatelet and anticoagulation drugs are paused prior to surgery [55]. In the case of bloody aspiration during puncture, the clinical course can be observed, surgery postponed if necessary, and bleeding complications can be promptly identified and treated. Following placement of CSF drainage, the patient is transferred to a normal ward. The spinal catheter is connected to a syringe pump and an infusion with 1 ml/h isotonic infusion solution is started in order to avoid blockage of the catheter. On the morning of surgery, the spinal catheter is checked for correct functioning and position, after which the patient is taken to the operating room. Once there, LiquoGuard®7, a system for continuous ICP monitoring and simultaneous pressure-guided CSF drainage, is connected.

An intraoperative ICP of less than 10 mm Hg is set as a target. In addition, the mean arterial blood pressure is elevated to 90 and 100 mm Hg after the implantation of the endoprothesis. These target parameters are maintained for 3 days postoperatively. CSF drainage is then paused, but the spinal catheter left in place in order to ensure a continuously monitoring of the intracranial pressure and to allow immediate CSF drainage in the case of neurological deficits. The spinal catheter is finally removed after additional 24 h.

Should paraplegia be observed postoperatively, CSF drainage is resumed for at least 7 days. The target ICP in this cases is below 7 mm Hg.

**Fig. 1:**
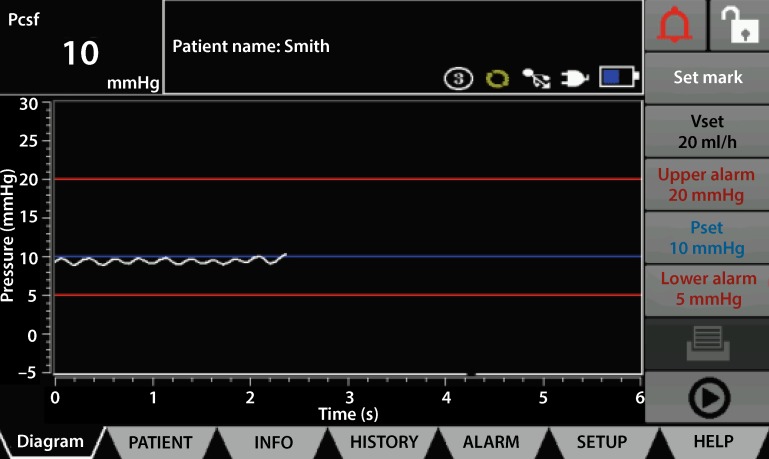
Automated cerebrospinal fluid (*CSF*) drainage using LiquoGuard®7, with continuous monitoring of intracranial pressure. Target pressure was set at 10 mm Hg (*Pset*) with alarm limits at 5 and 20 mm Hg. Current pressure is 10 mm Hg (*Pcsf*). If the current pressure exceeds the target pressure, CSF drainage at a maximum of 20 ml/h (*Vset*) begins and continues until the target pressure is achieved again. (With kind permission from the manufacturer, Möller Medical GmbH, Fulda, Germany)

Initial results with this standardized protocol using an automated, pressure-controlled system to monitor ICP and perform pressure-controlled CSF drainage by means of LiquoGuard®7 (Fig. 1) have been published, showing a spinal ischemia rate of 3% in a high-risk collective [47]. In total, a mean of 714 ml of CSF was drained per patient. The mean drainage volume per 24 h period was 192 ml. However, 33% of patients in this case series experienced complications caused either by the spinal catheter or by CSF drainage itself. These primarily included minor complications such as bloody fluid seen through the spinal catheter, CSF leakage requiring treatment, and postpuncture headache (29/30, 97% of complications). One patient died as a result of intracerebral hemorrhage (1/30, 3% of complications). No complications directly related to the use of the LiquorGuard®7 system were observed.

## Practical conclusion

- In addition to a multitude of invasive and noninvasive measures, perioperative CSF drainage during open surgical repair of the thoracic and thoracoabdominal aorta represents an effective means to reduce the incidence of spinal ischemia.
- Comparable evidence for its use in endovascular procedures is not yet available.
- Since CSF drainage itself can be associated with severe complications, its routine implementation in endovascular aortic procedures is not justified.
- Many centers use selective perioperative CSF drainage in patients at increased risk for spinal ischemia.
- Immediate CSF drainage combined with elevation of the mean arterial pressure represents an effective treatment for postoperative deficits due to spinal ischemia.
- The use of modern techniques, e. g., the LiquoGuard®7 system, can facilitate the management of CSF drainage.
